# Protective Factors Associated With Post-traumatic Outcomes in Individuals With Experiences of Psychosis

**DOI:** 10.3389/fpsyt.2021.735870

**Published:** 2021-11-29

**Authors:** Carolina Campodonico, Katherine Berry, Gillian Haddock, Filippo Varese

**Affiliations:** ^1^School of Psychology and Computer Science, University of Central Lancashire, Preston, Lancashire, United Kingdom; ^2^Complex Trauma and Resilience Research Unit, Greater Manchester Mental Health NHS Foundation Trust, Manchester Academic Health Science Centre, Manchester, United Kingdom; ^3^Division of Psychology and Mental Health, School of Health Sciences, Faculty of Biology, Medicine and Health, Manchester Academic Health Science Centre, The University of Manchester, Manchester, United Kingdom; ^4^Greater Manchester Mental Health NHS Foundation Trust, Manchester Academic Health Science Centre, Manchester, United Kingdom

**Keywords:** post-traumatic growth, resilience, PTSD, post-traumatic stress disorder, psychosis, trauma, CPTSD, PTG

## Abstract

Trauma and trauma-specific mental health difficulties (e.g., post-traumatic stress disorder) are highly prevalent in people with psychosis. However, not everyone develops post-traumatic symptoms, and some people even experience post-traumatic growth (PTG) following trauma. It is important to identify which protective factors are associated with less severe trauma symptoms and/or positive outcomes to inform the development and implementation of interventions fostering these variables. Eighty-five patients with experiences of psychosis took part in a cross-sectional study. They were administered questionnaires measuring exposure to traumatic events, symptoms of PTSD and complex PTSD and potential protective factors assumed to be associated with lower vulnerability for post-traumatic symptoms and higher post-traumatic growth (trait resilience, secure attachment, social support, adaptive coping, optimism, general self-efficacy). Multiple hierarchical regression showed that some of these protective factors, in particular optimism, were associated with lower post-traumatic symptoms, explaining 21% of the variance in complex PTSD symptoms and 16% of the variance in PTSD symptoms. However, the hypothesized protective factors, in particular resilience and adaptive coping, explained a considerably larger proportion of variance in PTG (44%). Our results suggest that whilst these variables provide only moderate protection from the vulnerability to experience post-traumatic stress, they may play an important role in allowing people to find meaning despite multiple traumas and subsequently lead more fulfilling lives. Therapies targeting the emotional and psychological consequences of trauma in people with psychosis might benefit from the integration of intervention strategies to enhance these additional psychological protective factors, which in turn may lead to positive treatment outcomes beyond the mere reduction of post-traumatic stress symptoms.

## Introduction

Trauma and trauma-related conditions such as post-traumatic stress disorder (PTSD) are highly prevalent among people with psychosis (1–3). Different relationships have been suggested to exist between trauma, PTSD, and psychosis. Trauma exposure, especially during childhood, can increase vulnerability to psychotic disorders or psychotic-like experiences such as delusions, hallucinations, disorganized thinking, and abnormal behavior (4–6). Furthermore, due to the phenomenological similarity between post-traumatic stress symptoms and specific symptoms of psychosis (e.g., flashbacks and hallucinatory experiences), it has been suggested that psychosis and PTSD could be part of the same spectrum of responses to traumatic events. The way these intrusive experiences are labeled determines the diagnostic interpretation of these symptoms as either a function of psychosis or PTSD (7, 8). Finally, psychotic symptoms or related experiences can be so distressing that they cause symptoms of PTSD—an idea captured with the term psychosis-related PTSD (9, 10).

PTSD is not the only adverse outcome that could follow exposure to traumatic events. In addition to PTSD, the International Classification of Diseases 11th edition manual (ICD-11) proposed the distinct diagnosis of complex PTSD (CPTSD), which is supported by an increasing number of studies (11, 12). CPTSD comprises both the core PTSD symptoms (re-experiencing, avoidance, and sense of threat), plus three additional symptom clusters collectively referred to as “Disturbances in Self Organization” (DSO) (13). These comprise affective dysregulation (i.e., difficulties in regulating emotions, which can manifest in heightened emotional arousal or feeling of numbness and dissociation), negative self-concept (i.e., persistent negative views of the self and extremely negative self-evaluations) and disturbed relationships (i.e., difficulties with developing and maintaining interpersonal relationships, which can result in feeling distant from others) (11). Despite the recognition of the co-occurrence of psychotic symptoms and CPTSD (14), the increasing evidence in support of the ICD-11 CPTSD diagnosis, and research suggesting that psychological constructs consistent with the DSO may be important mediators of the association between trauma and psychosis (15), research specifically focusing on CPTSD in psychosis is still lacking (3, 16).

While PTSD and CPTSD are two recognized negative outcomes that can follow a traumatic experience, research has shown the existence of protective factors that might instead lead to more positive outcomes. These outcomes refer to circumstances when individuals can use their social and personal characteristics to remain immune to the effects of trauma, to “bounce back” to previous levels of adjustment or to prosper and experience post-traumatic growth (PTG) (17). PTG is described as a positive change that people experience after a traumatic event, to the point of developing beyond their previous level of psychological functioning in certain areas of their lives (18). Furthermore, numerous meta-analyses and systematic reviews have been carried out to understand which factors seem to favor different positive outcomes following trauma in non-clinical populations. Social support (19–21), adaptive coping (21, 22), optimism (23, 24), trait resilience (25), self-efficacy (26, 27) and secure attachment (28, 29) are among the factors that seem to be more consistently associated to these positive post-traumatic outcomes.

Some research has been conducted on protective factors from trauma in people with psychosis, and PTG and its underpinnings are being increasingly investigated among service users (30). However, this has not been done as extensively as in other populations and never looking at numerous protective factors within the context of one study. When protective factors such as secure attachment were investigated, they were usually studied in association with their influence on psychotic symptoms [e.g., (31)]. In the rare instances where studies looked specifically at trauma and trauma-related responses in psychosis, research found that trait resilience (32) and adaptive coping (33) mediated the relationship between trauma and psychotic-like experiences, and that social support protected against the effects of trauma (34). To address the paucity of research on the topic, this present study investigated commonly recognized protective factors (social support, adaptive coping, optimism, trait resilience, self-efficacy and secure attachment) for three different post-traumatic outcomes in a sample of people with experiences of psychosis. First, we examined what protective factors were associated to different posttraumatic outcomes (PTSD, DSO and PTG), and then if these factors moderated the relationship between potentially traumatic life events and post-traumatic outcomes. It was hypothesized that: (1) participants with higher scores on the protective factors scales will report less severe symptoms of PTSD and DSO; (2) participants with higher scores on the protective factors scales will report higher scores on the PTG scale; (3) the protective factors will moderate the relationship between the amount of adverse event and PTSD and DSO symptoms severity.

## Materials and Methods

### Participants

Eighty-five participants were recruited from inpatient and outpatient services and from a list of eligible participants who had previously given consent to be contacted for research purposes by researchers at the authors' institution. Recruitment took place across five NHS Trusts in the North-West of England. Participants were considered eligible if they were: (1) aged 16 or above; (2) able to provide informed consent; (3) experiencing psychosis (e.g., having a diagnosis of schizophrenia) as confirmed with their mental health professional.

### Measures

A brief self-report demographic questionnaire was used to collect data on participants' gender, age, nationality, ethnicity, legal status, level of education, employment status, present mental health illnesses and treatment history.

The *Trauma And Life Events Checklist* [TALE; (35)] is a 22-item self-report measure specifically designed for routine trauma screening in psychosis services. It includes a list of common traumatic or stressful life events commonly reported by people with psychosis. For each event that is endorsed, participants are asked if the event occurred more than once and at which age(s). A total trauma exposure score can be derived by summing the number of items endorsed. Three additional items ask participants to identify which events are still affecting the person, as well as the extent of such impact using a scale from “not at all (0)” to “extremely (10).” Currently, the TALE is the only trauma checklist that includes psychosis-specific potentially traumatic events (e.g., traumatic reactions to psychotic symptoms, unusual behaviors or hospitalizations), and has shown moderate psychometric acceptability overall, with excellent reliability and convergent validity for sexual abuse (35).

The *International Trauma Questionnaire* [ITQ; (12)] is a self-report assessment tool to assess whether someone meets the criteria for ICD-11 PTSD and CPTSD. It consists of 12 items, 6 items measuring PTSD symptoms (re-experiencing, avoidance, and sense of current threat) and 6 items measuring DSO symptoms (negative self-concept, affective dysregulation, and disturbances in relationship). Impairment caused by PTSD and DSO symptoms is measured with three items each. Each item is rated on a 5-point Likert scale ranging from “Not at all” (0) to “Extremely” (4). In this study, ITQ responses were anchored on the traumatic event described as having the most significant impact on the person's life on the TALE. Possible scores for the PTSD and DSO symptoms subscales range from 0 to 24 and do not include the scores from the impairment items. The criteria for PTSD are met when each relevant symptom scores at least 2 (moderately), and when functional impairment is also observed (at least one of the three items scores ≥2). The criteria for possible CPTSD are met, when, in addition to PTSD, the participant also presents at least moderate scores across each DSO symptom, in conjunction with functional impairment. The ITQ has good psychometric and diagnostic properties and has shown to effectively capture the distinction between PTSD and CPTSD (12). Cronbach's α in this study was excellent for both PTSD 0.81 and DSO 0.89 scales.

The *Post-Traumatic Growth Inventory* [PTGI; (36)] is a 21-item scale that assesses positive changes occurring in response to major adversities. The items capture valued, positive changes resulting from the experience of trauma and adversity across several areas' of the person's life, including relating to others (e.g., “I have more compassion for others”), developing new possibilities (e.g., “I established a new path for my life”), personal strengths (e.g., “I know better that I can handle difficulties”), spiritual changes (e.g., “I have a stronger religious faith”), and appreciation for life (e.g., “I changed my priorities about what is important in life”). Items are scored on a 6-point Likert-type scale, from “I did not experience this change as a result of my crisis (0)” to “I experienced this change to a very great extent as a result of my crisis (5).” The total score, ranging from 0 to 105, is calculated by adding all the responses. The PTGI has satisfactory internal consistency in previous research (36). The reliability of the total PTGI scores was Cronbach's α = 0.88.

The *Connor-Davidson Resilience Scale* [CD-RISC; (37)] is a well-established measure of trait resilience that consists of 25 items. Each item is rated on a 5-point Likert scale ranging from “Not at all true” (0) to “True nearly all the time” (4). The total score ranges from 0–100, with higher scores reflecting greater trait resilience. Rating is based on how the respondent has felt during the past month. The CD-RISC demonstrates high construct validity, as well as high internal consistency (38). In this study, the Cronbach's α was 0.92.

The *Relationship Questionnaire* [RQ; (39)] is a self-report questionnaire that identifies four types of adult attachment styles (secure, dismissing, preoccupied, and fearful). In this study, the RQ was used to measure secure attachment, which is described by a paragraph depicting a confident attitude toward relationships in general. Participants are asked to rate their degree of correspondence with this paragraph on a 7-point scale, ranging from “Disagree strongly (1)” to “Agree strongly (7).” The RQ has shown to have good psychometric properties (39).

The *Multidimensional Scale of Perceived Social Support* [MSPSS; (40)] is a 12-item, self-report measure that evaluates support from family, friends and significant others, where “significant other” could be any person the participant feels particularly close to. The items are rated on a 7-point Likert-type scale with scores ranging from “very strongly disagree (1)” to “very strongly agree (7).” The MSPSS has demonstrated high internal consistency and convergent validity in a psychiatric sample (41). The scale has shown high internal reliability in this study with Cronbach's α = 0.93.

The *Brief Coping Orientation to Problems Experienced inventory* [Brief-COPE; (42)] is a 28-item self-report multidimensional inventory assessing 14 different forms of copying, each comprised of 2 items. Participants indicate whether they employed certain coping strategies based on a 4-point Likert scale ranging from “I haven't been doing this at all (1)” to “I've been doing this a lot (4).” Studies have grouped the original 14 coping scales into various overarching subscales. For this study, we used the adaptive coping subscale, which has been previously used in the psychosis literature (33, 43). Adaptive coping included active coping, planning, use of emotional support, use of instrumental support, positive reframing, religion, and humor items. The internal consistency in the present sample was good, with Cronbach's α = 0.78.

The *Life Orientation Test-Revised* [LOT-R; (44)] assesses individual trait differences in optimism. It contains 10 self-report items which ask respondents to indicate the degree to which they agree or disagree with six general statements, such as “In uncertain times, I usually expect the best,” “Overall, I expect more good things to happen to me than bad,” and “If something can go wrong for me it will (reverse-coded).” The LOT-R also comprises three positive, three negative and four filler items whose scores are not computed. Each item is rated on a 5-point Likert scale ranging from “strongly disagree (0)” to “strongly agree (4).” Higher scores indicate more life optimism. The psychometric properties of the LOT-R have been found to be satisfactory in non-clinical populations (45). The internal consistency in this study was high, with Cronbach's α = 0.90.

The *General Self-Efficacy Scale* [GSE; (46)] is a self-report measure of self-efficacy beliefs, with a focus on coping with hassles and stressful events. Respondents indicate their agreement with each of the ten items (e.g., “I can always manage to solve difficult problems if I try hard enough”) on a four-point Likert scale ranging from “Not at all true (1)” to “Exactly true (4),” and higher summated score represents better general self-efficacy among the participants. The GSE has shown high internal consistency coefficients for a variety of samples and countries (47). The Cronbach's α in the present study was 0.94.

The *Positive And Negative Syndrome Scale* [PANSS; (48)] was used to investigate if the severity of psychotic symptoms was a possible covariate in our analysis. Each item is rated on a seven-point severity scale ranging from “Absent (1)” to “Extreme (7)”. The PANSS assesses positive symptoms (e.g., delusions hallucinations) negative symptoms (e.g., blunted affect, emotional withdrawal) and general psychopathology (e.g., anxiety, depression) experienced by the person in the previous week. The PANSS is well established and validated measure of symptom severity in severe mental health problems and has shown to have adequate psychometric properties (49). The interviewer was trained to administer the PANSS and achieved an excellent overall reliability score against gold standard scores (ICC = 0.901). Queries concerning scoring were regularly discussed with the wider research team.

### Procedure

After receiving NHS and Health Research Authority ethics approval (reference number: 18/NW/0469), participants were recruited between May 2019 and December 2019. Testing took place in locations convenient to participants, including the participants' homes, hospitals, community mental health centers, and university premises. After providing informed written consent, participants were asked to complete the demographic form, the PANSS clinical interview and the battery of research questionnaires. Participants were then fully debriefed and received £10 as a thank you for participating.

### Data Analysis

Established procedures to identify multivariate outliers (combination of unusual scores on at least two variables) (50) found no evidence of influential cases in this dataset. As our variables were not normally distributed, non-parametric correlational analyses (Spearman's ρ) were carried out to examine the bivariate associations between the number of adverse life events, protective factors (adaptive coping, trait resilience, social support, secure attachment, optimism and general self-efficacy) and the outcome variables (PTSD, DSO and PTG). Following bivariate analyses, three multiple hierarchical regression models were conducted to examine whether protective factors predicted each of the post-traumatic outcomes (PTSD, DSO and PTG), and to investigate any increment in variation accounted for the addition of potential confounders (PANSS total, age, gender, and ethnicity). As PTSD and DSO were not normally distributed, the regression models including these dependent variables were bootstrapped. All regression analyses met the assumption of absence of multicollinearity (as confirmed by inspection of collinearity diagnostics, including variance inflation factor and tolerance statistics) and the assumption of normality of residuals. PROCESS (Model 1), a macro for SPSS, was then used to carry out an analysis to investigate if any of the protective factors moderated the relationship between the number of adverse events and outcome variables. All statistical analyses were carried out using SPSS version 25.

## Results

### Sample Characteristics

Demographic and descriptive measures for the current sample are displayed in Table 1. In summary, 60 participants were males and 25 were females, with a mean age of 44. Most of our participants had a diagnosis of schizophrenia (78%), followed by affective psychosis (14%) and psychosis NOS (8%). More than half of the participants reported clinically relevant post-traumatic symptoms on the ITQ (64%), with 13% meeting criteria for a diagnosis of PTSD, 29% meeting criteria for a diagnosis of CPTSD, and 22% meeting criteria for DSO symptoms (but not CPTSD).

**Table 1:** Demographic and clinical characteristics of the sample.

Age	Mean	44.0
	SD	13.2
Gender (%)	Male	70.6
	Female	29.4
Nationality (%)	British	96.4
	Irish	1.2
	French	1.2
	American	1.2
Ethnicity (%)	White	82.4
	Mixed	7.1
	Black	5.9
	Asian	3.5
	Other	1.2
Marital status (%)	Never married	70.6
	Married	17.6
	Divorced	9.4
	Separated	2.4
Education (%)	GCSE	36.5
	No qualification	32.9
	Graduated university	11.8
	A-levels	11.8
	Dropped out university	7.1
Employment (%)	Unemployed	82.4
	Working	7.1
	Studying	3.5
	Retired	7.1
Patient status (%)	Outpatient	72.9
	Inpatient	27.1
Diagnosis (%)	Schizophrenia	77.6
	Affective psychosis	14.1
	Psychosis NOS	8.2
Trauma group	No diagnosis	36.5
	CPTSD	28.2
	DSO	22.4
	PTSD	12.9
PANSS total	Mean	57.5
	SD	13.4

*CPTSD, complex post-traumatic stress disorder; DSO, disturbs in self-organization; GCSE, general certificate of secondary education; PANSS, positive and negative syndrome scale; Psychosis NOS, not otherwise specified; PTSD, post-traumatic stress disorder*.

All participants reported at least two different traumas and three participants reported up to seventeen different traumas out of the twenty included in the TALE. During childhood, 46% reported to have felt unsafe, unloved or unimportant, 31% reported to have experienced sexual abuse, and 20% reported to have gone thirsty, hungry or not have had a safe place to stay. Participants also reported to have often experienced potentially traumatic and highly distressing psychosis-related life events, with 85% reporting to have felt in danger or distress because of psychosis-related experiences, 62% reporting to have experienced a threatening or upsetting contact with mental health services, and 44% reporting acting in a way that put them or someone else in danger or that was strange or embarrassing.

### Correlational Analyses

Non-parametric correlational analyses are reported in Table 2. In partial support of our hypothesis, some but not all the protective factors were associated with less severe symptoms of PTSD and DSO. Optimism was associated with lower PTSD scores, whereas resilience, optimism and self-efficacy were associated with lower DSO scores. Contrary to predictions, adaptive coping was associated with *higher* PTSD symptoms. To better understand this unexpected association, we carried out additional correlation analyses considering the specific subscales of the adaptive coping measure and PTSD, and we found that planning (*r* = 0.409, *p* < 0.001), positive reframing (*r* = 0.220, *p* = 0.043) and religion (*r* = 0.254, *p* = 0.019) were the only subscales positively related to PTSD scores. In partial support of our hypothesis, participants with higher scores on some of the protective factor scales reported higher scores on the PTGI scale. PTG had a significant positive association with adaptive coping, resilience, optimism, and general self-efficacy. Contrary to predictions, secure attachment was not correlated with any post-traumatic outcome. The correlational analysis also showed a relationship between the number of potentially traumatic events as reported in the TALE, and PTSD and DSO. However, no association was found between number of traumatic events and PTG.

**Table 2:** Spearman's ρ correlations.

	**PANSS**	**PTSD**	**DSO**	**PTGI**	**Adaptive coping**	**Trait resilience**	**Social support**	**Secure attachment**	**Optimism**	**GSE**
TALE	0.189	0.290^**^	0.303^**^	−0.040	0.137	−0.024	0.013	−0.185	−0.091	−0.025
PANSS		0.304^**^	0.311^**^	−0.273^*^	0.121	−0.179	−0.361^**^	−0.080	−0.239^*^	−0.175
PTSD			0.556^**^	0.057	0.268^*^	−0.133	−0.041	−0.005	−0.324^**^	−0.157
DSO				−0.218^*^	0.000	−0.386^**^	−0.114	−0.082	−0.487^**^	−0.388^**^
PTG					0.422^**^	0.662^**^	0.388^**^	0.179	0.488^**^	0.446^**^
Adaptive coping						0.315^**^	0.331^**^	−0.042	0.173	0.165
Trait resilience							0.261^*^	0.112	0.695^**^	0.755^**^
Social support								0.198	0.163	0.200
Secure attachment									0.056	0.127
Optimism										0.574^**^

* *p < 0.05;*

** *p < 0.01*.

*DSO, disturbances in self-organization; GSE, general self-efficacy; PANSS, positive and negative syndrome scale; PTG, post-traumatic growth; PTSD, post-traumatic stress disorder; TALE, trauma and life events checklist*.

### Regression Analyses

Three separate hierarchical regressions were carried out with post-traumatic outcomes (PTSD, DSO and PTG) as the dependent variables while controlling for psychotic symptoms (PANSS Total) and demographic variables (age, gender, and ethnicity) (Table 3). The first hierarchical regression was carried out with PTSD as the dependent variable. After entering all the protective factors (adaptive coping, resilience, social support, secure attachment, optimism and GSE) in the first step of the analysis, the model explained 16% of the variance in the PTSD outcome [*F*_(6, 78)_ = 3.74, *p* = 0.003, *R*^2^ = 0.22, $R_{\text{Adjusted}}^{2}$ = 0.16]. The inclusion of psychotic symptoms in the second step did not significantly improve the prediction of PTSD (*R*^2^ = 0.23, significance of *R*^2^ change *p* = 0.35). In step three, the demographic variables did not contribute to the prediction of PTSD (respectively *R*^2^ = 0.24, significance of *R*^2^ change *p* = 0.96). Throughout the three steps of the regression, adaptive coping and optimism remained significant. Adaptive coping was positively associated with PTSD symptoms (I: β = 0.383, *p* = 0.001; II: β = 0.351, *p* = 0.003; III: β = 0.356, *p* = 0.004), while optimism was negatively associated with PTSD symptoms (I: β = −0.380, *p* = 0.006; II: β = −0.363, *p* = 0.010; III: β = −0.353, *p* = 0.015).

**Table 3:** Summary of the regression analyses.

	**PTSD**				**DSO**				**PTG**			
**Model**	**Predictor**	***SE***	**β**	***Sig*.**	**Predictor**	***SE***	**β**	***Sig*.**	**Predictor**	***SE***	**β**	***Sig*.**
1	Adaptive coping	0.087	0.383	0.001	Adaptive coping	0.090	0.119	0.267	Adaptive coping	0.263	0.223	0.015
	Trait resilience	0.066	0.069	0.702	Trait resilience	0.068	−0.089	0.614	Trait resilience	0.199	0.559	0.000
	Social support	0.589	−0.116	0.291	Social support	0.611	−0.062	0.562	Social support	1.777	0.152	0.093
	Secure attachment	0.361	0.029	0.776	Secure attachment	0.375	−0.046	0.649	Secure attachment	1.089	0.100	0.238
	Optimism	0.146	−0.380	0.006	Optimism	0.151	−0.400	0.003	Optimism	0.439	0.070	0.531
	General self-efficacy	0.139	−0.040	0.798	General Self-Efficacy	0.144	−0.079	0.597	General self-efficacy	0.418	−0.147	0.247
2	Adaptive coping	0.091	0.351	0.003	Adaptive coping	0.094	0.075	0.498	Adaptive coping	0.269	0.281	0.003
	Trait resilience	0.066	0.068	0.709	Trait resilience	0.068	−0.091	0.603	Trait resilience	0.194	0.562	0.000
	Social support	0.633	−0.076	0.521	Social support	0.653	−0.006	0.957	Social support	1.864	0.077	0.412
	Secure attachment	0.361	0.028	0.787	Secure attachment	0.373	−0.048	0.633	Secure attachment	1.064	0.103	0.216
	Optimism	0.147	−0.363	0.010	Optimism	0.152	−0.376	0.005	Optimism	0.433	0.038	0.727
	General self-efficacy	0.139	−0.041	0.792	General self-efficacy	0.143	−0.081	0.588	General self-efficacy	0.409	−0.145	0.243
	PANSS total	0.055	0.104	0.350	PANSS total	0.057	0.144	0.182	PANSS total	0.162	−0.193	0.032
3	Adaptive coping	0.095	0.356	0.004	Adaptive coping	0.096	0.081	0.478	Adaptive coping	0.263	0.320	0.001
	Trait resilience	0.068	0.079	0.675	Trait resilience	0.069	−0.046	0.796	Trait resilience	0.190	0.513	0.001
	Social support	0.685	−0.094	0.460	Social support	0.697	−0.051	0.673	Social support	1.887	0.040	0.674
	Secure attachment	0.374	0.019	0.856	Secure attachment	0.380	−0.060	0.558	Secure attachment	1.039	0.077	0.340
	Optimism	0.153	−0.353	0.015	Optimism	0.155	−0.355	0.010	Optimism	0.430	0.069	0.524
	General self-efficacy	0.144	−0.048	0.766	General self-efficacy	0.146	−0.119	0.435	General self-efficacy	0.397	−0.100	0.408
	PANSS total	0.057	0.099	0.391	PANSS total	0.058	0.115	0.294	PANSS total	0.159	−0.157	0.075
	Gender	1.572	0.057	0.599	Gender	1.598	0.126	0.226	Gender	4.371	0.037	0.648
	Age	0.055	−0.009	0.935	Age	0.056	0.042	0.689	Age	0.152	−0.244	0.004
	Ethnicity	1.876	−0.017	0.877	Ethnicity	1.907	−0.088	0.394	Ethnicity	1.637	0.026	0.750

*DSO, disturbances in self-organization; PANSS, positive and negative syndrome scale; PTG, post-traumatic growth; PTSD, post-traumatic stress disorder*.

A second regression model was estimated with DSO as the dependent variable. When entering the protective factors in step one, this model explained 21% of variance [*F*_(6, 78)_ = 4.79, *p* < 0.001, *R*^2^ = 0.27, $R_{\text{Adjusted}}^{2}$ = 0.21]. The inclusion of PANSS total in step two (*R*^2^ = 0.29, significance of *R*^2^ change *p* = 0.18) and of gender, age and ethnicity in step three (*R*^2^ = 0.31, significance of *R*^2^ change *p* = 0.48) did not significantly improve the prediction of DSO. Optimism was the only variable significantly associated with lower DSO symptoms across the three steps of the analysis (I: β = −0.400, *p* = 0.003; II: β = −0.376, *p* = 0.005; III: β = −0.355, *p* = 0.010).

The last hierarchical regression was conducted with PTG as the dependent variable, and in step one the model explained 44% of the variance [*F*_(6, 78)_ = 12.00, *p* < 0.001, *R*^2^ = 0.48, $R_{\text{Adjusted}}^{2}$ = 0.44]. In step one, resilience (β = 0.559, *p* < 0.001) and adaptive coping (β = 0.223, *p* = 0.015) predicted higher PTG scores. Adding PANSS total in step two significantly improved the prediction of PTG (*R*^2^ = 0.51, significance of *R*^2^ change *p* = 0.03). In step two adaptive coping (β = 0.281, *p* = 0.003) and resilience (β = 0.562, *p* < 0.001) were still significant predictors; furthermore, it was found that PANSS total was negatively associated with growth (β = −0.193, *p* = 0.032). Step three also showed a small significant improvement in the prediction of PTG (*R*^2^ = 0.57, significance of *R*^2^ change *p* = 0.03). Adaptive coping (β = 0.320, *p* = 0.001) and resilience (β = 0.513, *p* = 0.001) remained significant in step three, whereas PANSS was no longer a significant predictor. Age predicted lower PTG scores (β = −0.244, *p* = 0.004).

### Moderation Analysis

A series of moderation analyses were carried out using PROCESS (Model 1) to investigate if any of the protective factors moderated the relationship between the number of adverse life events and post-traumatic outcomes. The results of the analyses (shown in Appendix A) revealed that none of the interaction terms were significant.

## Discussion

This study investigated potential protective factors (social support, adaptive coping, optimism, trait resilience, self-efficacy, and secure attachment) and their relationship with post-traumatic outcomes (PTSD, DSO and PTG) in a sample of people experiencing psychosis. We hypothesized that the potential protective factors would be associated with less severe trauma symptoms and higher levels of PTG. We also hypothesized that the protective factors would moderate the relationship between number of adverse life events and post-traumatic outcomes. Correlational analyses showed that PTSD was negatively associated with optimism, DSO was negatively associated with resilience, optimism and general self-efficacy and PTG had a significant positive association with adaptive coping, resilience, optimism and general self-efficacy. Regression analyses carried out for each post-traumatic outcome while controlling for psychotic symptoms and demographic variables showed that adaptive coping predicted higher PTSD symptoms, that optimism negatively predicted both PTSD and DSO symptoms and that resilience and adaptive coping predicted higher PTG. The series of moderation analyses found that none of the protective factors moderated the relationship between adverse life events and PTSD and DSO symptoms.

Our results, in line with previous studies, indicated that participants had experienced multiple traumas and reported high rates of post-traumatic symptoms, with 13% of the sample meeting possible criteria for PTSD and 28% meeting criteria for CPTSD. Twenty-two percent of the sample presented potentially clinically significant DSO scores, despite not meeting criteria for CPTSD. Considering the high levels of DSO symptoms in this sample (across people meeting CPTSD criteria and not), our results suggest that people with psychosis might be more likely to experience complex trauma and particularly prone to develop DSO symptoms, thus supporting the need for more research on complex trauma and psychosis (3). It is also worth noting that most participants reported “psychosis-related traumas” (85% reported being scared by symptoms, 44% reported behaving in dangerous or embarrassing ways and 62% reported being upset from a contact with a mental health service). These results are consistent with previous studies highlighting high rates of psychosis-related trauma and associated post-traumatic stress in psychosis (9, 10). Despite the high rates of psychosis-related trauma, none of these events could be used as index traumas in this study (i.e., the event participants selected when reporting levels of traumatic stress on the ITQ scale). This was because in all cases these traumas were still ongoing and, as the authors of the TALE suggest, it would have been difficult to use ongoing events as a clear anchor for a post-traumatic symptoms assessment (35). Not being able to use psychosis-related traumas highlights the difficulty of assessing PR-PTSD using current PTSD diagnostic tools and suggesting that rates of PTSD and DSO symptoms may be even higher in people experiencing psychosis. It is worth noting that the TALE was developed for clinical purposes, and although it is a highly acceptable measure for people with psychosis, it does not instruct on how to consider the impact of recurrent or ongoing traumatic experiences. The measure, however, has great potential and might be a more sensible way to approach future research about trauma in psychosis, compared to trauma measures that do not capture elements that are specific for this population (i.e., PR-PTSD) (35).

In our analyses, optimism was consistently associated with lower levels of both PTSD and DSO symptoms, even after controlling for potential confounds. These findings support the results of a recent meta-analysis which found that optimism is associated with lower posttraumatic stress symptoms, possibly because it facilitates positive coping and adaptive response to obstacles (51). Longitudinal evidence has also suggested that individuals with high levels of optimism may be less prone to develop post-traumatic symptoms like avoidance and numbing because their optimist beliefs about their ability to cope with threats provide them with higher distress tolerance (52). Although the direction of influence between optimism and traumatic responses is still not clear, our results point at the potential importance of optimism as a protective factor from trauma in psychosis, and to the value of increasing it through targeted interventions (53). Counterintuitively, we found that adaptive coping predicted higher PTSD scores, despite being previously recognized as an important protective factor from post-traumatic outcomes (22), and being measured using a scale widely used with people with psychosis (43). Some items of the Brief-COPE (particularly those on the planning subscale i.e. “I've been trying to come up with a strategy about what to do” and “I've been thinking hard about what steps to take”) could substantially overlap with important processes involved in the maintenance of post-traumatic difficulties, such as avoidance and rumination, which have been shown to be key factors in the maintenance of PTSD symptoms (54). It is worth noting that the religion subscale (i.e., “I've been trying to find comfort in my religion or spiritual beliefs” and “I've been praying or meditating”) was also positively associated with PTSD symptom severity. However, the Brief-COPE only assesses to what extent a participant has adopted a specific coping strategy, and not if they benefited from it. It is possible that participants who tried to find comfort in their religion and failed might have felt hopeless, and might have experienced more traumatic symptoms as a consequence. The positive relationship between PTSD and hopelessness is well documented in the literature (55). This suggests that well-established measures of coping may not be always suitable in the context of assessing adapting coping in response to trauma. Also, prospective studies investigating the effects of religiosity on PTSD report mixed findings, ranging from no effect on symptoms to either improving or worsening (56), resulting in doubts on the effects of religiosity on PTSD.

When we investigated PTG, the results of the correlational and regression analysis showed that resilience and adaptive coping were positively associated with PTG even after controlling for confounds. It is important to note that while resilience and PTG both encompass the idea of thriving in adversity, they are generally regarded as distinct concepts, as resilience refers to positive adaptation despite significant adversity (57), while PTG suggests that people become stronger as a consequence of a trauma (18). Structural equation models have been used to illustrate that trait resilience is an important predictor of PTG (58) and it has been suggested that resilience plays a role in PTG as traumatic experiences may be less traumatic to resilient individuals (59). Supporting our findings, structural equation modeling has also shown that higher levels of adaptive coping predicts higher levels of PTG (60). As growth can coexist with the negative effects of trauma (61), adaptive coping might prove more useful in providing a framework to achieve a sense of control over adversity, which is necessary to experience PTG (62), rather than in protecting from traumatic symptoms. The regression analysis for PTG also suggested that the experience of growth might be negatively affected by the severity of psychotic symptoms, supporting the idea that psychotic symptoms are associated with a range of negative long-term functioning outcomes (63). Previous research on PTG in military personnel found some evidence that age can predict lower PTG scores (64) and suggests that the longer people live, the more likely they might be to experience growth.

Many hypothesized protective factors were not significantly related to any trauma outcomes, suggesting that they may be less important in understanding trauma responses in people with psychosis compared to other samples. Different factors might be more important in predicting trauma outcomes in people with experiences of psychosis, such as the content of voices and unusual beliefs. Furthermore, people with psychosis frequently present risk factors that could potentially interfere with these protective factors. For example, paranoid thoughts and consequent withdrawal from social contact could lead to isolation and prevent people from accessing social support (65). In this study, 15% of our participants could not identify anyone to whom they felt close to when completing the MSPSS, and 6% could only think of a member of their care team when asked who they felt they could rely on. Large social networks are important for improving global functioning in schizophrenia (66), and particularly having a partner helps lower levels of distress (67). In our sample, 22% indicated their romantic partner as their significant other, while 42% chose a family member, 13% chose a friend and 2% chose a religious guide. The relevance of having someone to whom we feel close to is further confirmed by the fact that 22% of our sample indicated “losing a close person” as the most traumatic event they experienced.

Certain protective factors, such as secure attachment, might not have emerged as protective for trauma because they are not very prevalent in people with psychosis. In our sample, 35% agreed to any extent (i.e., mildly, strongly, or very strongly) with the paragraph of the RQ which describes a secure attachment style. These results are not surprising considering that insecure attachment is pervasive in people with psychosis and directly links to psychotic vulnerability especially for paranoia (68, 69). Furthermore, secure attachment is associated with good social support (70) and a positive view of the self (71), both of which might have been lower in our sample, as highlighted by the high levels of DSO symptoms. Having a small sample, along with high levels of isolation, might have prevented social support and secure attachment from being recognized as significant protective factors in our analyses.

The fact that resilience was not found to be consistently protective from trauma is not entirely surprising. While the Connor-Davidson Resilience Scale (37) is recognized as a golden standard measure of resilience and is widely used in research, this tool specifically assesses trait resilience, and does not capture more recent conceptualizations that suggest that positive post-traumatic outcomes result from the combination of social and personal characteristics (17). Our results corroborate the idea that resilience might be better understood in terms of a collection of variables that allow individuals to successfully manage (i.e., cope with) the consequences of trauma, rather than simply as a trait. The results of the moderation analyses also showed that the hypothesized protective factors did not statistically moderate the association between the number of traumatic events and post-traumatic outcomes. However, we suggest that this might be due to our sample which was relatively small and highly traumatized.

This study has important implications as it suggests that interventions aiming to instill hope and optimism in service users, such as hearing stories of recovery or peer supporters who have recovered, might be used in dealing with post-traumatic symptoms. Such intervention might have the additional benefit of increasing hope in mental health staff, which would eventually benefit service users as positive professional expectancies have been recognized to affect patients' hopefulness (72). It is worth noting that most of the investigated protective factors, especially optimism, are already targeted in interventions such as the Cognitive Behavioral Therapy for Psychosis (CBTp), which examines how psychosocial dynamics can reduce the distress associated with the symptoms of psychosis and improve functioning (73, 74). There is strong evidence on the efficacy of CBTp (75), and our findings serve as a reminder of how this intervention could aim at enhancing some of these factors to ultimately facilitate recovery. Our results could also inform trauma therapies such as Trauma-focused Cognitive Behavior Therapy (TF-CBT) and Eye Movement Desensitization and Reprocessing (EMDR), which not only are safe and effective in improving traumatic symptoms in people with psychosis (76, 77), but can improve psychotic symptoms as well (78). According to TF-CBT after a patient processed their trauma and associated traumatic losses, they can start rebuilding life by restoring relationships and returning to pursuing one's aspirations (79), which relates to the proposed protective factor of social support and self-efficacy. Similarly, phase-based multimodal approaches for the treatment of CPTSD greatly focus on creating new meaning (80), which is at the base of the perception of gains necessary to experience growth (81). Our findings stress the importance of working on protective factors rather than on risk factors only and highlight the importance of recognizing post-traumatic growth as a viable post-traumatic outcome that clients could be helped to achieve with adequate support. Including the PTGI in routine clinical assessments, discussing growth with clients and focusing advice, resources and effort on supporting growth as well as recovery, would constitute important steps to support patients' wellbeing. Furthermore, clinicians should also explore the presence of DSO symptoms as these might be more relevant to this population in terms of post-traumatic reactions.

This study has some limitations. As our research was cross-sectional in nature, the results cannot be used to define the direction of influence among the variables investigated. The relatively small and heterogeneous sample limits generalisability, and the fact that participants relied on retrospective recall of childhood and adult trauma might have introduced some recall bias. Future research needs to be conducted with larger samples and longitudinal analyses, which would provide a more robust test for the direction of influence between variables. Studies need to account for the possible moderating effect of risk factors, such as levels of isolation, loneliness and medications. Further studies should examine which factors protect from trauma and foster growth in psychosis, investigating variables more specific for this group, such as therapeutic alliance and positive relationship with voices, both related to more positive outcomes in psychosis (82, 83).

In conclusion, our results highlight the importance of optimism as a protective factor against negative post-traumatic outcomes such as PTSD and CPTSD. Our findings suggest that protective factors such as resilience and adaptive coping might be less important in terms of protecting against negative outcomes, and more important in terms of enabling people to live a meaningful life despite multiple traumas. By focusing on fostering these protective factors, rather than simply reducing factors involved in the maintenance of distressing symptoms, future interventions may lead to a broader range of positive outcomes for trauma survivors who struggle with psychosis.

## Data Availability Statement

The raw data supporting the conclusions of this article will be made available by the authors, without undue reservation.

## Ethics Statement

The studies involving human participants were reviewed and approved by NHS and Health Research Authority Ethics Approval (Reference Number: 18/NW/0469). The patients/participants provided their written informed consent to participate in this study.

## Author Contributions

CC was responsible for data collection, data analysis, and drafting the original manuscript. FV and KB supervised CC during all stages of the research. All authors contributed to the design of this study and approved the final manuscript.

## Funding

This study was supported by a doctoral research grant from the University of Manchester. The sponsors had no role in the study design, collection, analysis, or interpretation of the data, or the preparation and approval of the manuscript.

## Conflict of Interest

The authors declare that the research was conducted in the absence of any commercial or financial relationships that could be construed as a potential conflict of interest.

## Publisher's Note

All claims expressed in this article are solely those of the authors and do not necessarily represent those of their affiliated organizations, or those of the publisher, the editors and the reviewers. Any product that may be evaluated in this article, or claim that may be made by its manufacturer, is not guaranteed or endorsed by the publisher.
