# Supplementary material for: Protective Factors Associated With Post-traumatic Outcomes in Individuals With Experiences of Psychosis
Source: Front Psychiatry. 2021 Nov 29;12:735870. doi: 10.3389/fpsyt.2021.735870 (PMC8666594; doi:10.3389/fpsyt.2021.735870)
Supplement: Supplementary file 1 [file Table_1.DOCX]

| **Appendix A.** Moderation analyses | | | | | | | | | | | | | | | |
| --- | --- | --- | --- | --- | --- | --- | --- | --- | --- | --- | --- | --- | --- | --- | --- |
|  | **PTSD** | | | |  | **DSO** | | | |  | **PTG** | | | | |
|  | *F(3, 81) = 4.71, p = .004, R^2^ =*   *.15* | | | |  | *F(3, 81) = 3.72, p = .015, R^2^ = .12* | | | |  | *F(3, 81) = 6.48, p = .001, R^2^ =*   *.19* | | | | |
|  | *β* | *se* | *t* | p |  | *β* | *se* | *t* | p |  | *β* | *se* | *t* | p |  |
| TALE | .73 | .77 | .95 | .34 | TALE | 1.74 | .84 | 2.09 | .04 | TALE | .89 | 2.76 | .32 | .75 |  |
| Adaptive coping | .27 | .19 | 1.38 | .17 | Adaptive coping | .21 | .21 | 1.01 | .31 | Adaptive coping | 1.61 | .69 | 2.34 | .02 |  |
| Int_1 | -.01 | .02 | -.39 | .70 | Int_1 | -.03 | .02 | -1.42 | .16 | Int_1 | -.03 | .07 | -.51 | .61 |  |
|  |  |  |  |  |  |  |  |  |  |  |  |  |  |  |  |
|  | *F(3, 81) = 3.59, p = .017, R^2^ = .12* | | | |  | *F(3, 81) = 9.10, p < .001, R^2^ = .25* | | | |  | *F(3, 81) = 16.63, p < .001, R^2^ = .38* | | | | |
|  | *β* | *se* | *t* | p |  | *β* | *se* | *t* | p |  | *β* | *se* | *t* | p |  |
| TALE | -.24 | .62 | -.38 | .71 | TALE | .68 | .61 | 1.11 | .27 | TALE | -2.26 | 1.92 | -1.18 | .24 |  |
| Resilience | -.15 | .10 | -1.60 | .11 | Resilience | -.14 | .09 | -1.46 | .15 | Resilience | .48 | .30 | 1.64 | .11 |  |
| Int_1 | .01 | .01 | 1.25 | .22 | Int_1 | .00 | .01 | -.19 | .85 | Int_1 | .04 | .03 | 1.23 | .22 |  |
|  |  |  |  |  |  |  |  |  |  |  |  |  |  |  |  |
|  | *F(3, 81) = 5.90, p = .001, R^2^ = .18* | | | |  | *F(3, 81) = 12.59, p < .001, R^2^ = .32* | | | |  | *F(3, 81) = 6.28, p = .001, R^2^ = .19* | | | | |
|  | *β* | *se* | *t* | p |  | *β* | *se* | *t* | p |  | *β* | *se* | *t* | p |  |
| TALE | .07 | .38 | .19 | .85 | TALE | .62 | .37 | 1.68 | .10 | TALE | -.29 | 1.37 | -.21 | .83 |  |
| Optimism | -.64 | .30 | -2.17 | .03 | Optimism | -.46 | .29 | -1.60 | .11 | Optimism | 1.38 | 1.09 | 1.27 | .21 |  |
| Int_1 | .04 | .03 | 1.23 | .22 | Int_1 | -.01 | .03 | -.30 | .77 | Int_1 | .04 | .11 | .34 | .74 |  |
|  |  |  |  |  |  |  |  |  |  |  |  |  |  |  |  |
|  | *F(3, 81) = 3.20, p = .028, R^2^ = .11* | | | |  | *F(3, 81) = 7.62, p < .001, R^2^ = .22* | | | |  | *F(3, 81) = 3.20, p = .028, R^2^ = .11* | | | | |
|  | *β* | *se* | *t* | p |  | *β* | *se* | *t* | p |  | *β* | *se* | *t* | p |  |
| TALE | .77 | .77 | 1.00 | .32 | TALE | .37 | .77 | .48 | .63 | TALE | -1.57 | 2.76 | -.57 | .57 |  |
| General self-efficacy | -.03 | .27 | -.11 | .91 | General self-efficacy | -.41 | .27 | -1.49 | .14 | General self-efficacy | .75 | .98 | .77 | .44 |  |
| Int_1 | -.01 | .03 | -.36 | .72 | Int_1 | .01 | .03 | .26 | .80 | Int_1 | .06 | .10 | .58 | .56 |  |
|  |  |  |  |  |  |  |  |  |  |  |  |  |  |  |  |
|  | *F(3, 81) = 2.66, p = .054, R^2^ = .09* | | | |  | *F(3, 81) = 3.37, p = .023, R^2^ = .11* | | | |  | *F(3, 81) = 4.22, p = .008, R^2^ = .14* | | | | |
|  | *β* | *se* | *t* | p |  | *β* | *se* | *t* | p |  | *β* | *se* | *t* | p |  |
| TALE | .88 | .77 | 1.14 | .26 | TALE | .61 | .82 | .74 | .46 | TALE | .83 | 2.78 | .30 | .77 |  |
| Social support | .50 | 1.47 | .34 | .73 | Social support | -.66 | 1.56 | -.42 | .67 | Social support | 8.86 | 5.29 | 1.67 | .10 |  |
| Int_1 | -.08 | .15 | -.50 | .62 | Int_1 | -.01 | .16 | -.04 | .97 | Int_1 | -.17 | .54 | -.32 | .75 |  |
|  |  |  |  |  |  |  |  |  |  |  |  |  |  |  |  |
|  | *F(3, 81) = 2.63, p = .056, R^2^ = .09* | | | |  | *F(3, 81) = 3.29, p = .025, R^2^ = .11* | | | |  | *F(3, 81) = 2.28, p = .085, R^2^ = .08* | | | | |
|  | *β* | *se* | *t* | p |  | *β* | *se* | *t* | p |  | *β* | *se* | *t* | p |  |
| TALE | .47 | .41 | 1.13 | .26 | TALE | .98 | .44 | 2.24 | .03 | TALE | -2.79 | 1.53 | -1.83 | .07 |  |
| Secure attachment | .02 | 1.02 | .02 | .98 | Secure attachment | .96 | 1.08 | .89 | .37 | Secure attachment | 3.78 | 3.78 | -1.46 | .15 |  |
| Int_1 | .02 | .11 | .17 | .87 | Int_1 | -.12 | .11 | -1.07 | .29 | Int_1 | .84 | .39 | 2.16 | .03 |  |
